# Effects of Replacing Soybean Meal With Degossypolized Cottonseed Protein on the Growth Performance, Protein Metabolism, Digestive Capacity, and Antioxidant Capacity of Hybrid Fish Hefang Bream

**DOI:** 10.1155/anu/4633901

**Published:** 2025-12-26

**Authors:** Qiuxin Yan, Xianyong Bu, Yongtao Liu, Chuanwei Yao, Zhen Wang, Manman Shi, Zhihao Zhang, Jinze Zhang, Junzhi Zhang, Jianlong Du, Yi Hu, Yueru Li, Kangsen Mai, Qinghui Ai

**Affiliations:** ^1^ Key Laboratory of Aquaculture Nutrition and Feed (Ministry of Agriculture and Rural Affairs) and Key Laboratory of Mariculture (Ministry of Education), Ocean University of China, 5 Yushan Road, Qingdao, 266003, Shandong, China, ouc.edu.cn; ^2^ Hunan Engineering Research Center for Utilization of Characteristics of Aquatic Resources, Hunan Agricultural University, Changsha, 410128, China, hunau.edu.cn

**Keywords:** amino acid profile, antioxidant capacity, degossypolized cottonseed protein, hybrid bream, liver health

## Abstract

An 8‐week feeding trial was conducted to evaluate the feasibility of substituting soybean meal (SBM) with degossypolized cottonseed protein (DCP) on Hefang bream. Five iso‐nitrogenous and iso‐lipidic diets were formulated with graded replacement levels of SBM by DCP at 0% (control), 25% (DCP25), 50% (DCP50), 75% (DCP75), and 100% (DCP100). Results showed that DCP could effectively replace up to 50% of dietary SBM in Hefang bream, without adverse influence on survival rate and growth performance. However, the specific growth rate in fish fed diets with DCP replacing 75% and 100% of SBM decreased significantly. Muscle essential amino acid profiles showed marked reductions in isoleucine, leucine, and lysine concentrations in fish fed diets with DCP replacing 75% and 100% of SBM, correlating with suppressed mTOR pathway transcription. The intestinal villi length and the intestinal trypsin activity in fish fed diets with DCP replacing 75% and 100% of SBM were significantly lower than those in the control group. The total antioxidant capacity and catalase activity in fish fed the diet with DCP replacing 100% of SBM were significantly lower than those in the control group. The mRNA expression of hepatic inflammatory cytokines in fish fed the diet with DCP replacing 25% of SBM was the lowest among the groups. In conclusion, these findings suggest that DCP can replace up to 50% of SBM in diets of the Hefang bream without compromising the growth performance. However, excessive dietary DCP (75%–100%) can induce protein utilization impairment, digestive dysfunction, oxidative stress, and hepatic inflammation.

## 1. Introduction

The expansion of global aquaculture has spurred a marked escalation in the need for aquatic feed. As a plant‐sourced protein, soybean meal (SBM) is extensively utilized as an optimal protein source in aquatic diets [1]. However, there are many limiting factors for the application of SBM in feed. The various antinutritional factors (such as soybean antigenic proteins and trypsin inhibitors) contained in SBM can compromise the integrity of piscine intestinal mucosa, diminish nutrient assimilation efficiency, and provoke enteric inflammatory disorders [2, 3]. Meanwhile, insufficient supply has led to an increase in SBM prices [4–6]. These constraints have catalyzed the exploration of nontraditional protein matrices, particularly noncompetitive with human food chains [7, 8]. Considering the easy availability and protection of human food, sustainable nongrain protein sources such as degossypolized cottonseed protein (DCP) have become one of the best choices.

DCP, alternatively termed cottonseed protein concentrate (CPC), serves as a high‐value nutritional ingredient derived through the reduction of free gossypol (FG) in cottonseed meal to below the safety threshold (<400 mg/kg) through physical or chemical treatment coupled with microbial fermentation while retaining a well‐balanced amino acid profile [9, 10]. Prior investigations revealed that strategic integration of DCP as a fish meal (FM) or alternative in aquafeeds could enhances growth parameters, mitigate oxidative damage, and elevate digestive efficacy in aquatic animals. For instance, replacing 20% of FM with DCP in diets improved the growth performance of large yellow croaker (*Larimichthys crocea*) [11]. Besides, partial replacement of 15% SBM with CPC in diets augmented antioxidant capacity in grass carp (*Ctenopharyngodon idellus*) [12]. Moreover, incorporating up to 15% CPC to replace FM in *Litopenaeus vannamei* diets increased the intestinal α‐amylase activity, trypsin activity, and lipase activity, indicating improved digestive enzyme activity [13]. Collectively, these studies suggest that DCP (15%–20%) can effectively replace FM or SBM in aquafeeds, promoting the growth and health of diverse aquatic animals.

Previous studies have demonstrated that herbivorous Hefang bream (optimal dietary protein levels: ~30%) exhibited high protein content, high unsaturated fatty acids, and low carbohydrates, making it an important hybrid bream for the sustainable aquaculture industry [14–17]. The SBM remains the principal dietary protein component in formulated feeds for Hefang bream currently, while no studies were conducted on the application of DCP as a substitute for SBM in diets of the Hefang bream. Therefore, this study evaluated the effectiveness of using DCP as an alternative to SBM in Hefang bream. The results provide actionable insights for incorporating DCP into aquafeed of herbivorous fish.

## 2. Materials and Methods

### 2.1. Ethics Statement

The research adhered rigorously to the Guiding Principles for Laboratory Animal Management (Chinese Order No. 676 of the State Council, Revised 1 March 2017).

### 2.2. Diets Formulation

Five experimental diets were formulated with isonitrogen (~33% crude protein) and isolipid (~4% crude lipid) The control group was a reference diet formulated with 50% SBM as the primary protein source. The DCP was incorporated into the control formulation at incremental substitution levels (0%, 25%, 50%, 75%, and 100%) to replace SBM, and named DCP0, DCP25, DCP50, DCP75, and DCP100 (Table 1). During diets preparation, ingredients were ground to 80‐mesh fineness and uniformly integrated with soybean oil following the formulation. A measured volume of water was subsequently introduced to achieve optimal hydration, followed by extrusion processing using a pelletizing apparatus to generate uniform cylindrical feed particles of 2.0 mm diameter. The moist pellets were then dried at 40°C for 12 h and stored at ‐20°C until use.

**Table 1:** Formulation and proximate composition of experimental diets for Hefang bream (% dry matter).

Ingredients	Diets^4^				
	DCP0	DCP25	DCP50	DCP75	DCP100
Fish meal^1^	5.00	5.00	5.00	5.00	5.00
Soybean meal^1^	50.00	37.50	25.00	12.50	0.00
Degossypolized cottonseed protein^2^	0.00	8.40	16.80	25.20	33.60
Rapeseed meal^1^	8.00	8.00	8.00	8.00	8.00
Wheat bran^1^	2.00	2.00	2.00	2.00	2.00
Wheat flour^1^	23.00	23.00	23.00	23.00	23.00
Rice bran^1^	4.00	4.00	4.00	4.00	4.00
α‐Starch^1^	0.90	4.40	8.40	12.30	16.30
Soybean oil^1^	3.60	4.20	4.30	4.50	4.60
Calcium phosphate^1^	2.50	2.50	2.50	2.50	2.50
Premix^3^	1.00	1.00	1.00	1.00	1.00
Proximate composition	—	—	—	—	—
Crude protein	33.69	33.71	33.84	33.59	33.34
Crude lipid	4.66	4.61	4.55	4.54	4.54
Ash	7.87	7.52	7.44	7.34	7.17
Moisture	6.51	6.49	6.62	6.58	6.64

^1^Ingredients were purchased from Qingdao Master Biotech Co., Ltd; Fish meal (crude protein:67.48%, crude lipid:8.22%), Soybean meal (crude protein:42.49%, crude lipid:1.55%).

^2^Degossypolized cottonseed protein (crude protein: 65.34%, crude lipid: 0.29%, free gossypol: 228 mg/kg) was acquired from Xinjiang Jinlan Plant Protein Co., Ltd. (Xinjiang, China).

^3^Premix is provided by Qingdao Master Biotechnology Co., Ltd., composition (kg^−1^ of diet): Vitamin A acetate 300,000 IU; Riboflavin 800 mg; Folic acid 150 mg; Magnesium 10,000 mg; Copper 400 mg; Vitamin D3 100,000 IU; Pyridoxine hydrochloride 600 mg; D‐biotin 4 mg Iron 12,000 mg; Cobalt 30 mg; d1‐α‐tocopheryl acetate 2400 mg; Cyanocobalamin 1.5 mg; L‐ascorbyl‐2‐phosphate 7000 mg; Zinc 5000 mg; Selenium 10 mg; Manganese 2000 mg; Iodine 100 mg; Thiamine nitrate 500 mg; Niacinamide 2500 mg; Inositol 8000 mg.

^4^DCP0 served as the control group, while DCP25, DCP50, DCP75, and DCP100 were formulated by replacing 25%, 50%, 75%, and 100% of the soybean meal in the control group.

### 2.3. Feeding Trial

The Hefang bream were sourced from Changsha Wangcheng Hechi Agricultural Development Co., Ltd., a commercial aquaculture supplier located in Hunan Province, China. The fertile hybrid lineage (BTF1‐BTF6) established through blunt snout bream (*Megalobrama amblycephala*, BSB, ♀) × topmouth culter (*Culter alburnus*, TC, ♂) by the distant hybridization. Then, the Hefang bream was obtained by BSB (♀) × the first‐round backcross offspring (female BTF1 × male BSB, ♂) [18–20]. Following a 7‐day acclimation with diets of control group, 600 juvenile hybrid breams (initial body weight 8.01 ± 0.02 g) were allocated into 15 polyethylene cages (2.0 × 2.0 × 2.0 m), with triplicate groups assigned to each dietary treatment and 40 fish per replicate. The fish received thrice‐daily feedings (06:00, 12:00, 17:30) at a ration equivalent to 3%–5% of their body weight. Aquatic environmental parameters remained within optimal thresholds throughout the trial period to ensure physiological stability, with temperature (25.00 ± 4.50°C), dissolved oxygen (6.70 ± 0.60 mg/L), ammonia nitrogen (≤0.30 mg/L), and pH (8.00 ± 0.20).

### 2.4. Sample Collection

The fish were fasted for 24 h following the 8‐week feeding trial, after which the eugenol was administered to the fish as an anesthetic agent at a 1:12,000 dilution. Within each experimental cage, the quantity and weight of the fish were documented to calculate the survival rate (SR), final body weight (FBW), and specific growth rate (SGR). Six fish were randomly selected from each net cage for whole‐fish proximate composition analysis. Another six fish were randomly chosen from each net cage for quantification of morphometric parameters (body length and weight) and physical indices (hepatopancreas weight and visceral weight), which were subsequently used to compute the hepatosomatic index (HSI), viscerosomatic index (VSI), and condition factor (CF). The muscle samples (harvested from the region above the lateral line of the head), intestinal tissues, and hepatic tissue were harvested from nine fish per experimental net cage and immediately stored at −80°C for further analysis. Then, to analyze morphological characteristics, intestinal samples from three fish per cage were fixed in a 4% paraformaldehyde solution.

### 2.5. Analysis of Proximate Composition and Amino Acids Concentrations

The proximate composition was assessed in accordance with established analytical protocols authorized by the Association of Official Analytical Chemists [21], involving the determination of crude lipid, crude protein, moisture, and ash content. Hepatic and muscular lipid concentrations were determined via a chloroform‐methanol solvent system (2:1 volume ratio), in accordance with Folch’s established extraction protocol [22].

Amino acid composition in diets (Table 2) and muscle tissue was determined. The samples were freeze‐dried using the Alpha1‐4LDplus freeze dryer (Christ, Germany), followed by acid hydrolysis (6 N HCl at 110°C for 22 h). Then, the analysis of amino acid concentrations was performed using an L‐8900 high‐performance automated analyzer (Hitachi High‐Technologies Corporation, Japan).

**Table 2:** Amino acid composition of experimental diets (% dry matter).

Amino acids	Diets^1^				
	DCP0	DCP25	DCP50	DCP75	DCP100
Threonine	1.16	1.38	1.30	1.21	1.28
Valine	1.02	1.21	1.16	1.11	1.18
Methionine	0.36	0.43	0.46	0.35	0.35
Isoleucine	1.10	1.26	1.18	1.05	1.13
Leucine	2.16	2.51	2.39	2.14	2.28
Phenylalanine	1.47	1.80	1.69	1.62	1.69
Lysine	1.60	1.80	1.77	1.59	1.66
Histidine	0.76	0.90	0.91	0.87	0.92
Arginine	1.88	2.55	2.80	2.92	3.13
ΣEAA	11.51	13.84	13.66	12.86	13.62
Aspartic acid	3.00	3.54	3.34	3.08	3.29
Serine	1.42	1.71	1.63	1.54	1.63
Glutamic acid	4.63	5.79	5.81	5.72	6.06
Glycine	1.53	1.82	1.75	1.65	1.75
Alanine	1.23	1.47	1.38	1.29	1.36
Cystine	0.36	0.45	0.46	0.42	0.43
Tyrosine	0.96	1.20	1.08	0.99	1.03
Proline	1.71	1.83	1.71	1.56	1.70
ΣNEAA	26.35	31.65	30.82	29.11	30.87
ΣTAA	37.86	45.49	44.48	41.97	44.49

*Note:* ΣEAA, total essential amino acids; ΣNEAA, total nonessential amino acids; ΣTAA, total amino acids.

^1^DCP0 served as the control group, while DCP25, DCP50, DCP75, and DCP100 were formulated by replacing 25%, 50%, 75%, and 100% of the soybean meal in the control group.

### 2.6. Histological Analysis of Intestinal Tissues

The middle intestine was sliced into 5 mm sections, followed by dehydration in a graded sequence of increasing concentrations of ethanol. Then, the samples were soaked into the xylene solution. The dehydrated samples were embedded within paraffin wax, sectioned into 6 μm sections using the RM2235 microtome (LEICA, Germany), and stained with hematoxylin and eosin (H&E). The tissue sections were examined and photographed using the CX31RTSF microscope (Olympus, Japan). Morphometric parameters of muscle layer thickness and intestinal villi dimensions (length and width) were analyzed through standardized digital image analysis employing ImageJ software. In each field of view, three well‐oriented, intact, and representative villi were selected for measurement.

### 2.7. Digestive Enzyme Activity and Antioxidant Capacity

Digestive enzyme activities and antioxidant parameters were assayed utilizing commercially available assay kits (Nanjing Jiancheng Bio‐engineering Institute, China). Specifically, digestive enzymes: trypsin (TPS), lipase (LPS), and α‐amylase (AMS). Antioxidants: total antioxidant capacity (T‐AOC), malondialdehyde (MDA), reduced glutathione (GSH), superoxide dismutase (SOD), catalase (CAT), and glutathione peroxidase (GSH‐PX). Sample preparation, tissue homogenization, and specific procedures were executed as per the manufacturer’s guidelines supplied with the assay kit to ensure accuracy and consistency.

### 2.8. Serum Biochemistry

Serum activities of alkaline phosphatase (AKP), alanine transaminase (ALT), and aspartate transaminase (AST), as well as the content of total protein (TP), total cholesterol (T‐CHO), triglycerides (TG), glucose (GLU), were determined by utilizing commercially available kits (Nanjing Jiancheng Bioengineering Institute, China) following the manufacturer’s guidelines.

### 2.9. Real‐Time PCR Analysis

The expression levels of genes related to protein metabolism in the liver and muscle, as well as those associated with hepatic inflammation, were measured. Total RNA isolation was performed with RNAiso Plus reagent (Takara, Japan), followed by cDNA synthesis using the PrimeScript™ RT kit (Takara, Japan). RT‐qPCR analyses were conducted on a CFX96™ thermocycler system (Bio‐Rad Laboratories, USA) to quantify target gene expression with fluorescence‐based detection. Since the genomic sequence of the Hefang bream has not been published, gene sequences were obtained by determining the transcriptome sequence of the Hefang bream, based on which the primers were engineered with Primer Premier 5.0 (Premier Biosoft, USA) (Table 3). The specificity of the products was determined by melting curve analysis. The amplification efficiency of the target genes was analyzed, ranging from 97% to 110%. *β*‐actin was utilized as the internal control. Transcript abundance was quantified and normalized through the 2^−ΔΔCt^ method to determine relative expression values [23].

**Table 3:** Sequence of primers used for real‐time PCR analysis.

Gene	Forward primer sequence (5′ to 3′)	Reverse primer sequence (5′ to 3′)
*tor*	GCAGCCACAGCAGTCCAATG	TTACAGCCACTACCAGCAGACC
*4ebp1*	CCGACCACCAGACGAATCACA	TGAGCGACAGCATCAGTACAGG
*4ebp2*	GCAATGTCGTCCAGTCGTCAG	CCTCCAGGAGTGGTGCAATAGT
*s6k1*	AGAACATCCGTCCAGAGTGCTT	GAGGTGTCCGAGAGCCATAGAG
*igf-1*	GTTGAAGCCATCAGCCAGCATT	TGAGTGTAGGCAGACCGATTCG
*tnf-α*	GCTGTCTGCTTCACGCTCAAC	AGCCTGGTCCTGGTTCACTCT
*il1-β*	ACGATAAGACCAGCACGACCTT	TCTCAGCGTCACAGCCATCAA
*il-6*	ATCAGCACGCCTCTCCTCAG	CTCCGTTGTCCACTCTTCCTCT
*nlrp3*	TGAGATTGGCTGGCTGTATGGT	GAGAGGCTGGACTCCTGATTGT
*il-10*	GGGCTTTCCTGTGAGGCTGAA	GCTGTTGGCAGAATGGTCTCC
*tgf-β*	ACGGAGGCTGTCAGTGAGTG	TCTGTCGGTGGCTCTTGTGTT
*arg1*	GGCAAGGTTGTGTTGTGAAGGA	GGCGTGGTCAAAGGTGTGTTT
*socs3*	TTCTACTGGAGCACCGTGAGC	GAAGGAGCAGGAATCGCATTGG
*β-actin*	TGTCCACCTTCCAGCAGATGT	GTCACCTTCACCGTTCCAGTT

*Note: tor*, target of rapamycin; *4ebp1*, eukaryotic translation initiation factor 4E binding protein 1; *4ebp2*, eukaryotic translation initiation factor 4E binding protein 2; *s6k1*, ribosomal protein S6 kinase 1; *igf-1*, insulin‐like growth factor 1; *tnf-α*, tumor necrosis factor‐α; *il-1*β, interleukin‐1β; *il-6*, interleukin‐6; *nlrp3*,nucleotide‐binding oligomerization domain‐like receptor protein 3*; il-10*, interleukin‐10; *tgf-β*, transforming growth factor‐β; *arg1*, arginase1; *socs3*, suppressor of cytokine signaling 3.

### 2.10. Calculations and Statistical Analysis

Survival rate %=Qf×100/Qi

Specific growth rate SGR, %/day=LnWf−LnWi×100/trial days

Protein efficiency ratio PER=Wf−Wi/Pi

Condition factor CF, g cm3=Wf×100/Lf3

Hepatosomatic index HSI, %=Wl×100/Wb

Viscerosomatic index VSI, %=Wv×100/Wb

where *Q*_*f*_, *Q*_*i*_, *W*_*f*_, *W*_*i*_, *L*_*f*_, *W*_*l*_, *W*_*b*_, *W*_*v*_, and *P*_*i*_ denote the final fish quantity, initial fish quantity, final fish weight, initial fish weight, final fish body length, liver weight, body weight, visceral weight, and total protein intake.

Statistical analyses were conducted using SPSS version 26.0 (IBM, USA). Normality and homogeneity of variance were first verified for the data. Intergroup differences were analyzed using one‐way ANOVA, followed by pairwise comparisons performed with Tukey’s test. Orthogonal polynomial contrast analysis was applied to assess linear and quadratic relationships in the data set. The significance level was set at *p* > 0.05 to indicate the presence of no significant differences. Data were presented as mean ± S.E.M (standard error of the mean).

## 3. Results

### 3.1. Survival, Growth Performance, and Physical Indexes

There were no significant differences in SR among the groups (*p* > 0.05) (Table 4). With increasing dietary DCP, the FBW and SGR declined progressively, consistent with the patterns described in both linear regression and quadratic models (Table 4). Compared to the control group, the FBW and SGR in fish fed diets with DCP replacing 75% and 100% of SBM were significantly lower (*p* < 0.05), while those in groups with 25% and 50% replacement showed no significant difference (*p* > 0.05). Besides, CF in fish fed diets with DCP replacing 25% and 50% of SBM was significantly reduced (*p* > 0.05). Moreover, substitution of SBM with DCP in the diet had no significant effect on HIS, VSI, and PER compared to the control group (*p* > 0.05).

**Table 4:** Survival rate, growth performance, and physical indexes of Hefang bream fed the experimental diets (means ± S.E.M.)^**1**^.

Parameters	Diets^2^					Orthogonal polynomial contrasts		
	DCP0	DCP25	DCP50	DCP75	DCP100	*p*‐Value^1^	Linear^1^	Quadratic^1^
IBW (g)	8.00 ± 0.01	8.01 ± 0.01	8.01 ± 0.01	8.03 ± 0.02	8.02 ± 0.03	0.805	0.337	0.573
FBW (g)	26.66 ± 0.26^a^	25.77 ± 0.25^ab^	25.76 ± 0.37^ab^	25.06 ± 0.24^b^	24.88 ± 0.52^b^	0.030	0.001	0.005
SR (%)	100.00 ± 0.00	100.00 ± 0.00	100.00 ± 0.00	100.00 ± 0.00	100.00 ± 0.00	—	—	—
SGR (%/d)	2.15 ± 0.02^a^	2.09 ± 0.02^ab^	2.08 ± 0.02^ab^	2.03 ± 0.02^b^	2.02 ± 0.04^b^	0.043	0.002	0.007
PER (%)	1.57 ± 0.022	1.49 ± 0.021	1.49 ± 0.030	1.44 ± 0.022	1.43 ± 0.046	0.052	0.003	0.009
CF (g/cm^3^)	2.15 ± 0.00^ab^	2.06 ± 0.02^c^	2.08 ± 0.01^c^	2.13 ± 0.01^b^	2.19 ± 0.01^a^	<0.001	0.120	<0.001
HSI (%)	2.28 ± 0.05^ab^	2.19 ± 0.02^ab^	2.11 ± 0.04^b^	2.36 ± 0.03^a^	2.37 ± 0.05^a^	0.004	0.104	0.015
VSI (%)	15.56 ± 0.09	15.2 ± 0.10	15.26 ± 0.17	15.6 ± 0.07	15.73 ± 0.19	0.068	0.163	0.030

Abbreviations: CF, condition factor; FBW, final body weight; HSI, hepatosomatic index; IBW, initial body weight; PER, protein efficiency ratio; SGR, specific growth rate; SR, survival rate; VSI, viscerosomatic index.

^1^Data are means of triplicate. According to Tukey’s multiple comparison test, values with same superscript letters within the same row indicate not significant differences (*p* > 0.05); *p*‐value represents the *p*‐value of one‐way ANOVA; Linear represents the *p*‐value of linear trend; Quadratic represents the *p*‐value of quadratic trend.

^2^DCP0 served as the control group, while DCP25, DCP50, DCP75, and DCP100 were formulated by replacing 25%, 50%, 75%, and 100% of the soybean meal in the control group.

### 3.2. Proximate Composition

The crude lipid content in both whole fish and muscle demonstrated an initial decrease succeeded by a subsequent increase as the dietary DCP substitution increased, following linear and quadratic models (Table 5). Compared to the control group, replacement of 25% SBM protein significantly reduced crude lipid content in both whole fish and muscle (*p* > 0.05). Increasing dietary DCP replacement resulted in a reduction in whole fish crude protein content, following linear and quadratic models. Fish fed diets with DCP replacing 75% and 100% of SBM had significantly lower whole‐fish crude protein content than those in the control group (*p* > 0.05). With increasing dietary DCP, the hepatic crude lipid content followed a significantly linear and quadratic increase with increasing dietary DCP (*p* > 0.05). The hepatic crude lipid content in fish fed diets with DCP replacing 50%, 75%, and 100% of SBM was significantly higher than that in the control group (*p* > 0.05). The whole‐fish ash content, muscle crude protein content, and moisture content (assessed in the whole fish, muscle, and liver) showed no significant differences between the treatment and control groups (*p* > 0.05).

**Table 5:** Proximate composition of Hefang bream fed the experimental diets (means ± S.E.M.)^**1**^.

Parameters	Diets^2^					Orthogonal polynomial contrasts		
	DCP0	DCP25	DCP50	DCP75	DCP100	*p*‐Value^1^	Linear^1^	Quadratic^1^
Whole fish								
Crude lipid (% w.w)	11.39 ± 0.06^c^	9.86 ± 0.09^d^	12.38 ± 0.2^b^	12.62 ± 0.18^ab^	13.20 ± 0.17^a^	<0.001	0.001	0.003
Crude protein (% w.w)	17.46 ± 0.09^a^	17.30 ± 0.1^a^	17.05 ± 0.06^ab^	16.83 ± 0.14^bc^	16.60 ± 0.06^c^	0.001	<0.001	<0.001
Moisture (%)	67.96 ± 0.91	69.56 ± 0.56	67.77 ± 1.25	66.69 ± 1.35	66.11 ± 1.31	0.299	0.078	0.152
Ash (% w.w)	5.45 ± 0.05	5.44 ± 0.07	5.63 ± 0.17	5.46 ± 0.08	5.35 ± 0.12	0.498	0.617	0.346
Muscle								
Crude lipid (% w.w)	2.90 ± 0.03^c^	2.72 ± 0.03^d^	2.75 ± 0.03^d^	3.27 ± 0.01^b^	3.81 ± 0.02^a^	<0.001	<0.001	<0.001
Crude protein (% w.w)	19.16 ± 0.35	19.11 ± 0.11	18.85 ± 0.17	18.66 ± 0.16	18.04 ± 0.34	0.055	0.003	0.007
Moisture (%)	77.85 ± 0.65^ab^	79.01 ± 0.54^a^	77.28 ± 0.2^ab^	76.90 ± 0.3^b^	76.75 ± 0.38^b^	0.029	0.021	0.063
Liver								
Crude lipid (% w.w)	15.34 ± 0.08^c^	16.39 ± 0.22^bc^	16.65 ± 0.13^b^	17.02 ± 0.28^b^	20.36 ± 0.42^a^	<0.001	<0.001	<0.001
Moisture (%)	62.64 ± 0.88	62.82 ± 0.77	61.44 ± 0.82	62.53 ± 1.34	59.27 ± 1.21	0.161	0.056	0.094

Abbreviation: w.w, wet weight.

^1^Data are means of triplicate. According to Tukey’s multiple comparison test, values with same superscript letters within the same row indicate not significant differences (*p* > 0.05); *p*‐value represents the *p*‐value of one‐way ANOVA; Linear represents the *p*‐value of linear trend; Quadratic represents the *p*‐value of quadratic trend.

^2^DCP0 served as the control group, while DCP25, DCP50, DCP75, and DCP100 were formulated by replacing 25%, 50%, 75%, and 100% of the soybean meal in the control group.

### 3.3. Amino Acid Profile and Gene Expression Related to Protein Metabolism in the Liver and Muscle

The amino acid profile indicated that all amino acids, except aspartic acid, exhibited a significant linear trend (*p* < 0.05) (Table 6). Specifically, in fish fed diets with DCP replacing 75% and 100% of SBM, the muscle levels of isoleucine, leucine, lysine, total amino acids, and total essential amino acids were significantly lower than those in the control group (*p* < 0.05). In fish fed diets with DCP replacing 75% and 100% of SBM, compared to those in the control group, the levels of serine, alanine, and proline (nonessential amino acids) were significantly reduced (*p* < 0.05). The treatment and control groups showed no significant differences in aspartic acid and tyrosine concentrations (*p* < 0.05).

**Table 6:** Amino acid composition of muscle in Hefang bream fed the experimental diets (% dry matter) (means ± S.E.M.)^**1**^.

Amino acids	Diets^2^					Orthogonal polynomial contrasts		
	DCP0	DCP25	DCP50	DCP75	DCP100	*p*‐Value^1^	Linear^1^	Quadratic^1^
Threonine	3.51 ± 0.18^a^	3.37 ± 0.11^ab^	3.36 ± 0.02^ab^	3.11 ± 0.12^ab^	2.88 ± 0.05^b^	0.017	<0.001	0.002
Valine	2.76 ± 0.10^a^	2.64 ± 0.07^a^	2.58 ± 0.01^ab^	2.43 ± 0.07^ab^	2.27 ± 0.08^b^	<0.001	<0.001	0.007
Methionine	2.02 ± 0.14^a^	1.81 ± 0.15^ab^	2.03 ± 0.02^a^	1.67 ± 0.06^ab^	1.41 ± 0.10^b^	0.004	0.006	0.009
Isoleucine	3.13 ± 0.15^a^	2.92 ± 0.09^ab^	2.91 ± 0.01^ab^	2.66 ± 0.11^bc^	2.44 ± 0.08^c^	<0.001	0.001	0.005
Leucine	6.88 ± 0.33^a^	6.38 ± 0.14^ab^	6.45 ± 0.04^ab^	5.84 ± 0.2^bc^	5.43 ± 0.15^c^	<0.001	<0.001	0.003
Phenylalanine	3.53 ± 0.13^ab^	3.69 ± 0.03^a^	3.28 ± 0.06^ab^	3.11 ± 0.09^b^	3.28 ± 0.2^ab^	0.022	0.072	0.036
Lysine	7.61 ± 0.33^a^	7.19 ± 0.13^ab^	7.15 ± 0.06^abc^	6.57 ± 0.2^bc^	6.26 ± 0.13^c^	<0.001	<0.001	0.004
Histidine	2.93 ± 0.15^a^	2.81 ± 0.05^a^	2.80 ± 0.04^ab^	2.59 ± 0.04^ab^	2.43 ± 0.05^b^	<0.001	0.001	0.008
Arginine	4.62 ± 0.20^a^	4.46 ± 0.17^a^	4.30 ± 0.02^ab^	4.05 ± 0.1^ab^	3.70 ± 0.13^b^	<0.001	<0.001	0.006
ΣEAA	36.98 ± 1.70^a^	35.27 ± 0.80^ab^	34.86 ± 0.19^abc^	31.81 ± 0.8^bc^	30.32 ± 0.98^c^	<0.001	0.001	0.006
Aspartic acid	6.97 ± 0.49	8.28 ± 0.27	8.35 ± 0.04	7.35 ± 0.45	7.07 ± 0.12	0.619	0.021	0.079
Serine	3.39 ± 0.12^a^	3.18 ± 0.06^ab^	3.14 ± 0.04^abc^	2.90 ± 0.12^bc^	2.77 ± 0.06^c^	<0.001	<0.001	0.004
Glutamic acid	10.58 ± 0.42^a^	10.01 ± 0.3^ab^	9.87 ± 0.09^ab^	8.98 ± 0.44^ab^	8.41 ± 0.39^b^	<0.001	0.001	0.010
Glycine	4.38 ± 0.24^ab^	4.55 ± 0.13^a^	3.90 ± 0.05^abc^	3.71 ± 0.13^bc^	3.57 ± 0.15^c^	<0.001	0.002	0.004
Alanine	4.64 ± 0.18^a^	4.50 ± 0.1^ab^	4.30 ± 0.04^ab^	4.00 ± 0.14^bc^	3.56 ± 0.14^c^	<0.001	<0.001	0.001
Cystine	0.68 ± 0.03^a^	0.62 ± 0.03^ab^	0.67 ± 0.06^a^	0.65 ± 0.01^a^	0.50 ± 0.01^b^	0.018	0.015	0.011
Tyrosine	2.78 ± 0.10	2.79 ± 0.02	2.65 ± 0.03	2.64 ± 0.05	2.58 ± 0.12	0.026	0.094	0.286
Proline	2.17 ± 0.04^a^	2.11 ± 0.03^ab^	2.02 ± 0.02^ab^	1.93 ± 0.04^b^	1.96 ± 0.07^b^	0.001	0.002	0.011
ΣNEAA	35.6 ± 0.62^ab^	36.05 ± 0.79^a^	34.91 ± 0.11^ab^	32.16 ± 1.23^bc^	30.42 ± 0.74^c^	<0.001	<0.001	0.002
ΣTAA	72.58 ± 2.32^a^	71.31 ± 1.59^ab^	69.77 ± 0.28^ab^	64.19 ± 2.18^bc^	60.52 ± 1.57^c^	<0.001	<0.001	0.003

*Note:* ΣEAA, total essential amino acids; ΣNEAA, total nonessential amino acids; ΣTAA, total amino acids.

^1^Data are means of triplicate. According to Tukey’s multiple comparison test, values with same superscript letters within the same row indicate not significant differences (*p* > 0.05); *p*‐value represents the *p*‐value of one‐way ANOVA; Linear represents the *p*‐value of linear trend; Quadratic represents the *p*‐value of quadratic trend.

^2^DCP0 served as the control group, while DCP25, DCP50, DCP75, and DCP100 were formulated by replacing 25%, 50%, 75%, and 100% of the soybean meal in the control group.

**Figure 1:**
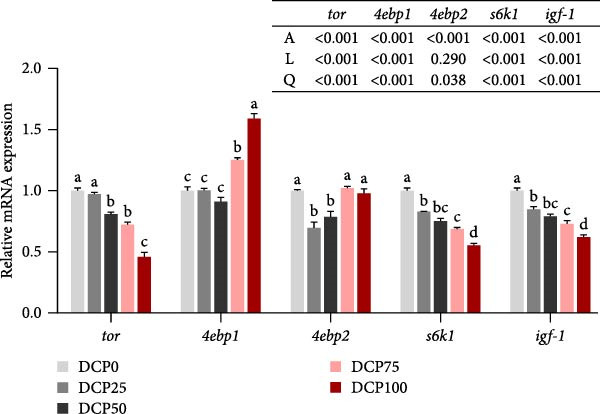
The expression of genes related to protein metabolism in liver of Hefang bream fed the experimental diets. *tor*, target of rapamycin; *4ebp1*, eukaryotic translation initiation factor 4E binding protein 1; *4ebp2*, eukaryotic translation initiation factor 4E binding protein 2; *s6k1*, ribosomal protein S6 kinase 1; *igf-1*, insulin‐like growth factor 1. Values are mean ± S.E.M. (*n* = 3). According to Tukey’s multiple comparison test, bars with same superscript letters indicate not significant differences (*p* > 0.05). A represents the *p*‐value of one‐way ANOVA; L represents the *p*‐value of linear trend; Q represents the *p*‐value of quadratic trend.

The expression of protein metabolism‐related genes (*tor*, *4ebp1*, *4ebp2*, *s6k1*, and *igf-1*) in the liver followed quadratic models with increasing dietary DCP (Figure 1). Compared to the control group, the gene expression of *tor*, *s6k1*, and *igf-1* in fish fed diets with DCP replacing 50%, 75%, and 100% of SBM was significantly downregulated (*p* < 0.05). In contrast, the gene expression of *4ebp1* expression in fish fed diets with DCP replacing 75% and 100% of SBM was significantly upregulated (*p* < 0.05), whereas *4ebp2* expression in fish fed diets with DCP replacing 25% and 50% of SBM was downregulated compared to the control group (*p* < 0.05).

**Figure 2:**
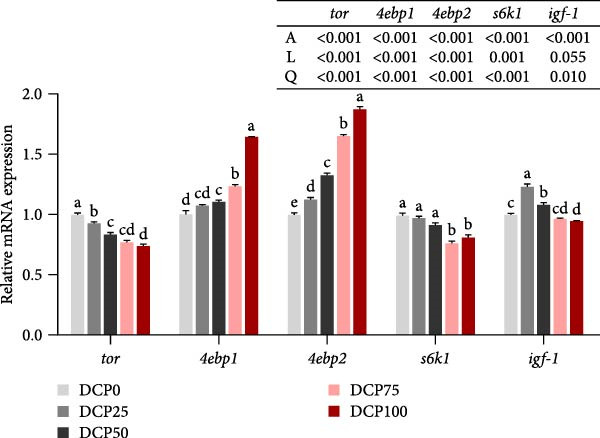
The expression of genes related to protein metabolism in muscle of Hefang bream fed the experimental diets. *tor*, target of rapamycin; *4ebp1*, eukaryotic translation initiation factor 4E binding protein 1; *4ebp2*, eukaryotic translation initiation factor 4E binding protein 2; *s6k1*, ribosomal protein S6 kinase 1; *igf-1*, insulin‐like growth factor 1. Values are mean ± S.E.M. (*n* = 3). According to Tukey’s multiple comparison test, bars with same superscript letters indicate not significant differences (*p* > 0.05). A represents the *p*‐value of one‐way ANOVA; L represents the *p*‐value of linear trend; Q represents the *p*‐value of quadratic trend.

In the muscle, with increasing dietary DCP supplementation in diets, a significant quadratic trend was observed in the expression of protein metabolism‐related genes (*tor*, *4ebp1*, *4ebp2*, *s6k1*, and *igf-1*) (*p* < 0.05) (Figure 2). Compared to the control group, the gene expression levels of *tor* and *s6k1* in fish fed diets with DCP replacing 75% and 100% of SBM were significantly downregulated (*p* < 0.05). Furthermore, fish fed diets with DCP replacing 50%, 75%, and 100% of SBM showed significant upregulation of *4ebp1* and *4ebp2* expression (*p* < 0.05). And the expression of *igf-1* was significantly upregulated in fish fed diets with DCP replacing 25% and 50% of SBM (*p* < 0.05).

### 3.4. Intestinal Morphology and Digestive Enzyme Activity

With increasing dietary DCP, mid‐intestinal villus length exhibited both linear and quadratic trends (Figure 3A, B). In fish fed diets with DCP replacing 50%, 75%, and 100% of SBM, mid‐intestinal villus length exhibited a significant reduction compared to the control group (*p* < 0.05), whereas there were no significant differences in villus width among the groups (Figure 3A, C, *p* > 0.05). In addition, fish fed diets with DCP replacing 25% and 50% of SBM exhibited a significantly thicker muscle layer compared to those in the control group (Figure 3A, D, *p* < 0.05).

Figure 3Histological morphology of Hefang bream fed the experimental diets. (A) Micromorphology of gut (scale bar = 100 μm), VL, length of villus; VW, villus width; MT, thickness of muscle layer; (B) length of villus; (C) villus width; (D) thickness of muscle layer. Values are mean ± S.E.M. (*n* = 3). According to Tukey’s multiple comparison test, bars with same superscript letters indicate not significant differences (*p* > 0.05). A represents the *p*‐value of one‐way ANOVA; L represents the *p*‐value of linear trend; Q represents the *p*‐value of quadratic trend.

**Figure:**
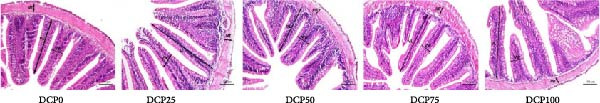
(A)

**Figure:**
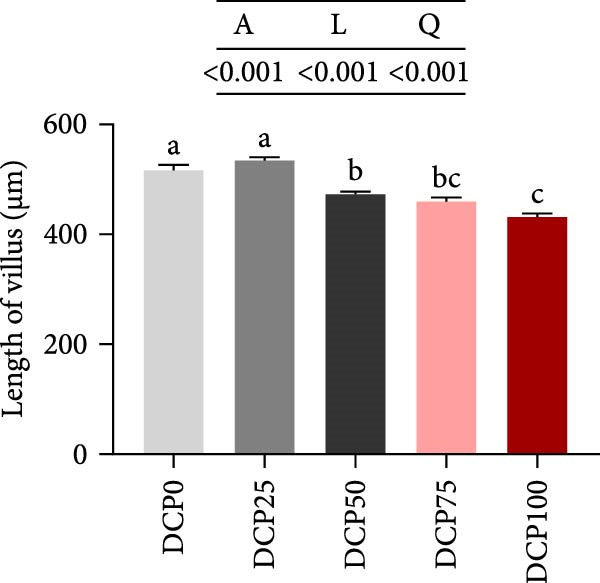
(B)

**Figure:**
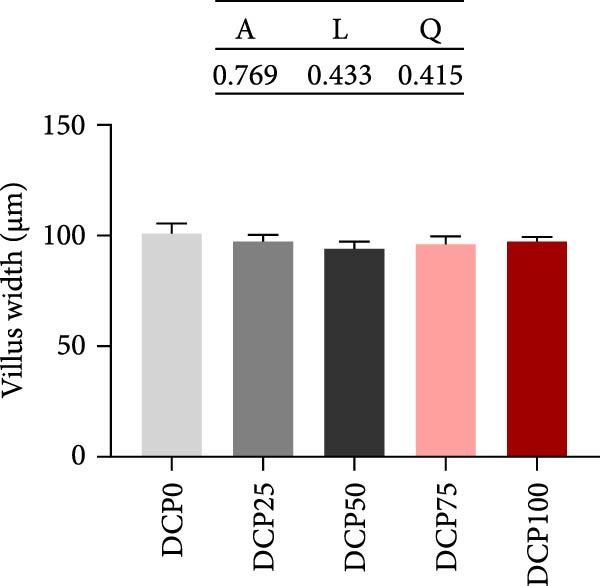
(C)

**Figure:**
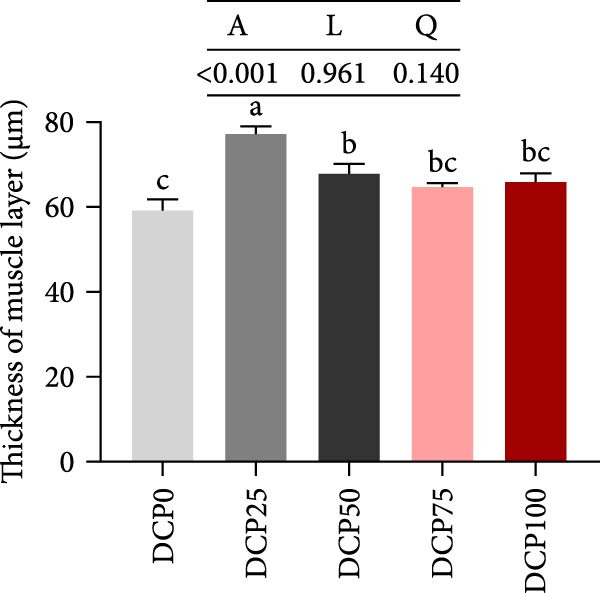
(D)

The activities of intestinal trypsin and the α‐amylase demonstrated a significant linear and quadratic trend (Figure 4). In fish fed diets with DCP replacing 75% and 100% of SBM, intestinal trypsin activity exhibited a significant reduction compared to the control group (*p* < 0.05). In contrast, intestinal α‐amylase activity was lowest in the control group compared to all treatment groups. However, there were no significant differences in intestinal lipase activity among the groups (Figure 4, *p* > 0.05).

Figure 4Intestine digestive enzyme activities of Hefang bream fed the experimental diets. (A) Trypsin; (B) lipase; (C) alpha‐amylase. Values are mean ± S.E.M. (*n* = 3). According to Tukey’s multiple comparison test, bars with same superscript letters indicate not significant differences (*p* > 0.05). A represents the *p*‐value of one‐way ANOVA; L represents the *p*‐value of linear trend; Q represents the *p*‐value of quadratic trend.

**Figure:**
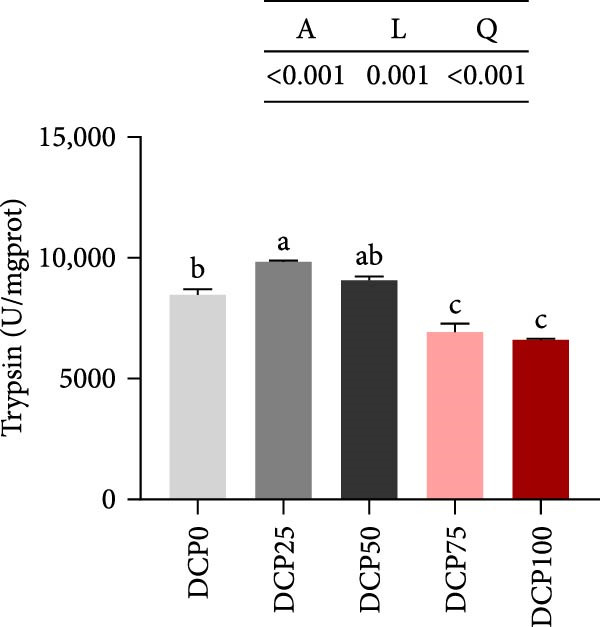
(A)

**Figure:**
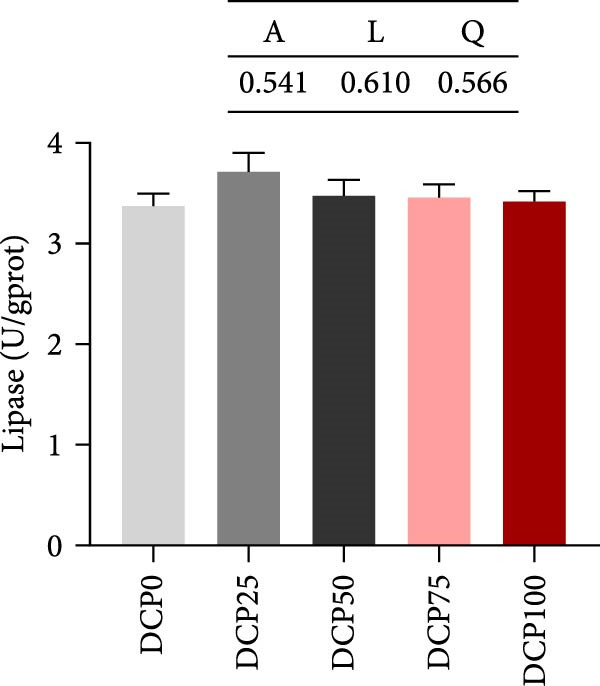
(B)

**Figure:**
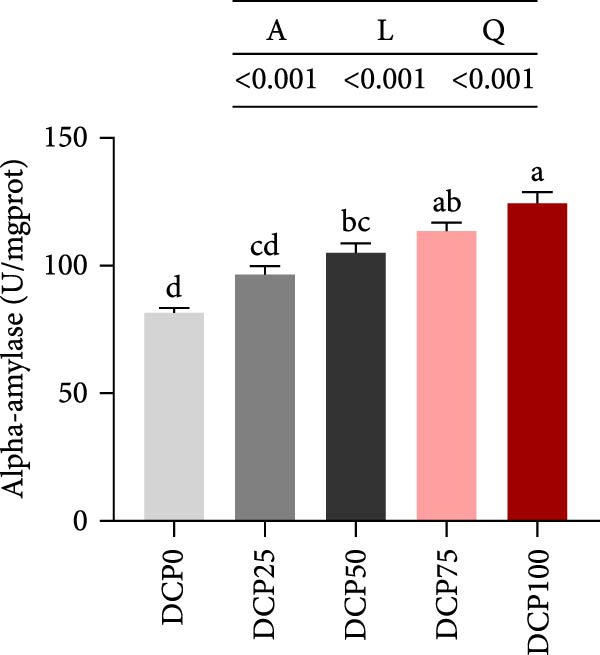
(C)

### 3.5. Antioxidant Capacity

With increasing dietary DCP, T‐AOC, GSH content, and CAT activity showed an initial increase succeeded by a subsequent decrease, following a quadratic trend, with all three parameters peaking at the 25% replacement level (Figure 5A, C, and E). In contrast, the MDA content initially decreased then increased with increasing dietary DCP supplementation, following a significant linear and quadratic trend (Figure 5B). Correspondingly, compared to that in the control group, fish fed the diet with DCP replacing 100% of SBM exhibited a significant increase in MDA levels (*p* < 0.05). The activities of SOD and GSH‐PX demonstrated a significant quadratic trend (*p* < 0.05), reaching peak levels in fish fed the diet with DCP replacing 50% of SBM (Figure 5D, F).

Figure 5Liver antioxidant capacity of Hefang bream fed the experimental diets. (A) Total antioxidant capacity; (B) malondialdehyde; (C) reduced glutathione; (D) superoxide dismutase; (E) catalase; (F) glutathione peroxidase. Values are mean ± S.E.M. (*n* = 3). According to Tukey’s multiple comparison test, bars with same superscript letters indicate not significant differences (*p* > 0.05). A represents the p‐value of one‐way ANOVA; L represents the *p*‐value of linear trend; Q represents the *p*‐value of quadratic trend.

**Figure:**
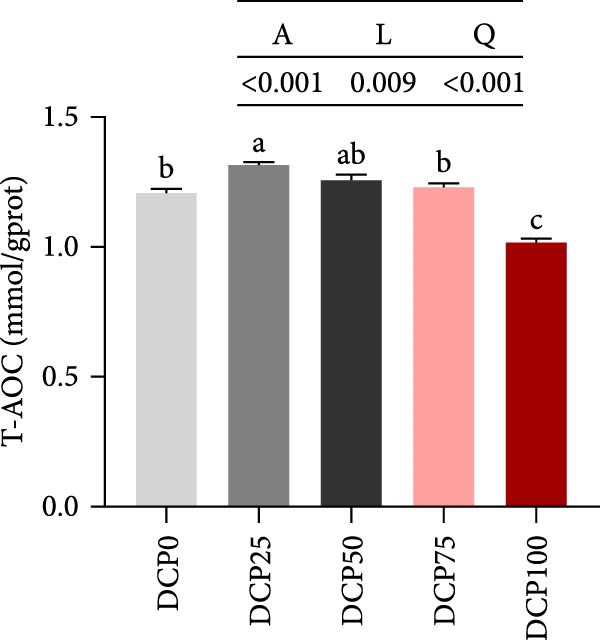
(A)

**Figure:**
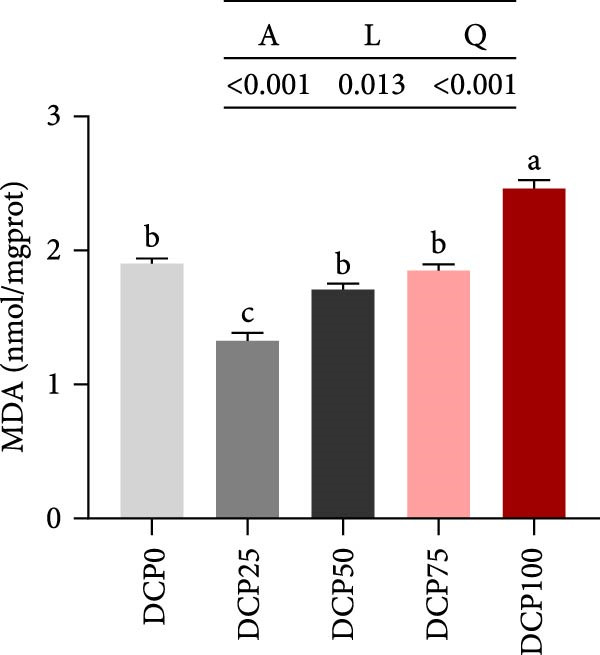
(B)

**Figure:**
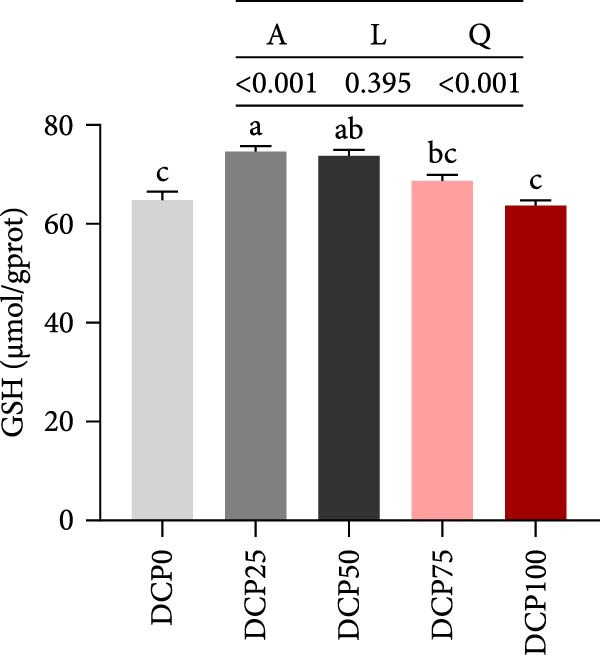
(C)

**Figure:**
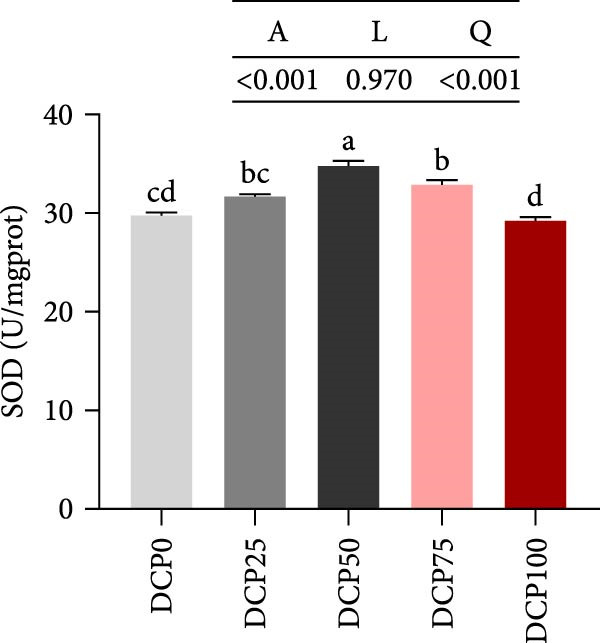
(D)

**Figure:**
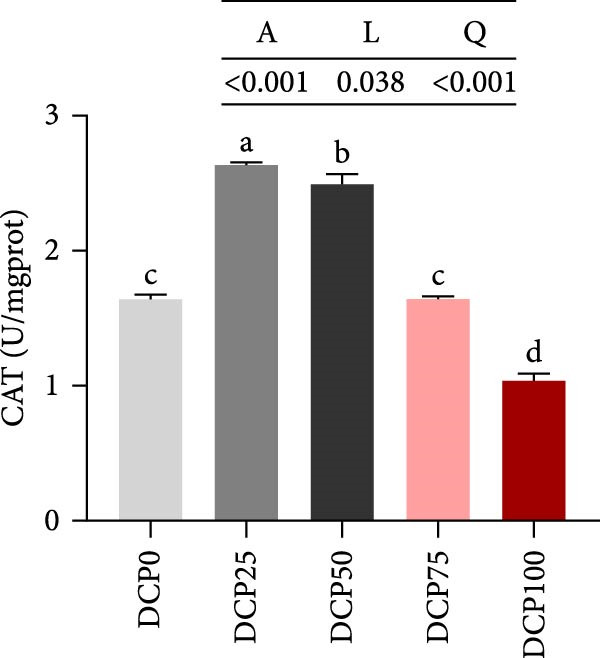
(E)

**Figure:**
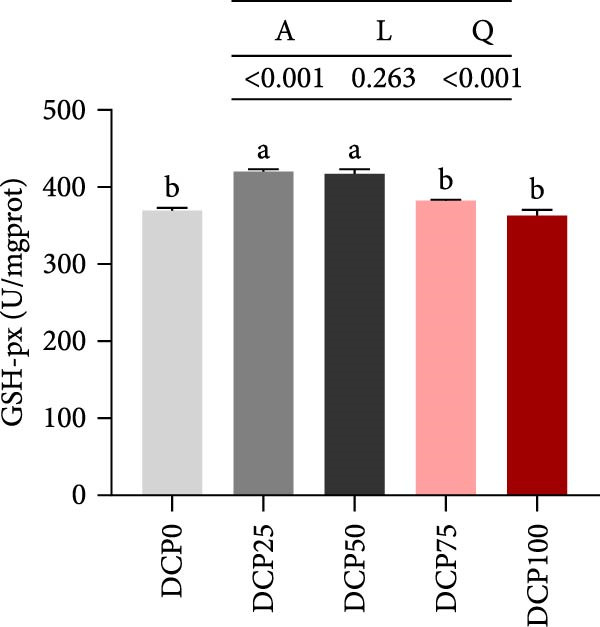
(F)

### 3.6. Serum Biochemical Profile

With increasing dietary DCP, the serum T‐CHO content showed a significant quadratic trend (Figure 6A). Fish fed diets with 50% and 75% SBM replaced by DCP exhibited significantly reduced serum T‐CHO levels compared to those in the control group (*p* < 0.05). TG and GLU levels exhibited both linear and quadratic variations (Figure 6B, G). In fish fed diets with DCP replacing 75% and 100% of SBM, TG and GLU levels were significantly higher than those in the control group (*p* < 0.05). Additionally, an increasing DCP replacement in diets resulted in a decline in TP content and AKP activity, with trends fitting both linear and quadratic models (Figure 6C, D). Compared to the control group, fish in all treatment groups exhibited significantly reduced TP levels and AKP activity (*p* < 0.05). Furthermore, serum ALT and AST activities showed significant linear and quadratic trends (Figure 6E, F) with increasing dietary DCP. Specifically, fish fed diets with 100% SBM replaced by DCP exhibited significantly elevated serum ALT and AST activities (*p* < 0.05).

Figure 6Serum biochemical indices of Hefang bream fed the experimental diets. (A) Total cholesterol; (B) triglycerides; (C) total protein; (D) alkaline phosphatase; (E) alanine transaminase; (F) aspartate transaminase; (G) glucose. Values are mean ± S.E.M. (*n* = 3). According to Tukey’s multiple comparison test, bars with same superscript letters indicate not significant differences (*p* > 0.05). A represents the *p*‐value of one‐way ANOVA; L represents the *p*‐value of linear trend; Q represents the *p*‐value of quadratic trend.

**Figure:**
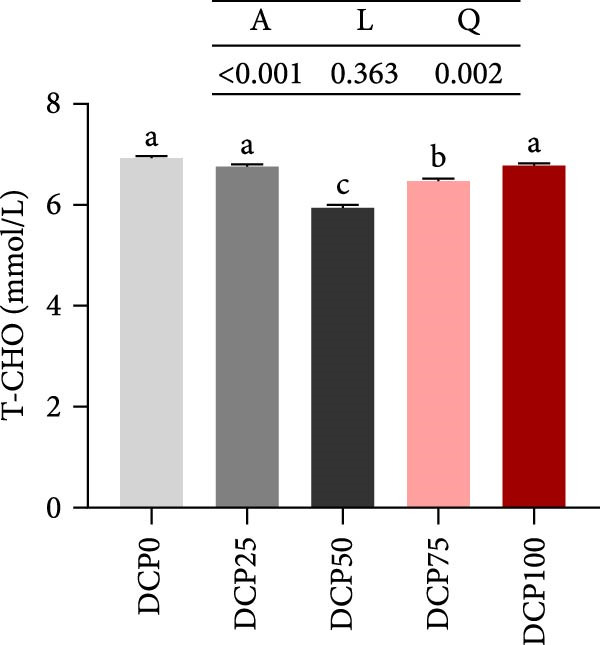
(A)

**Figure:**
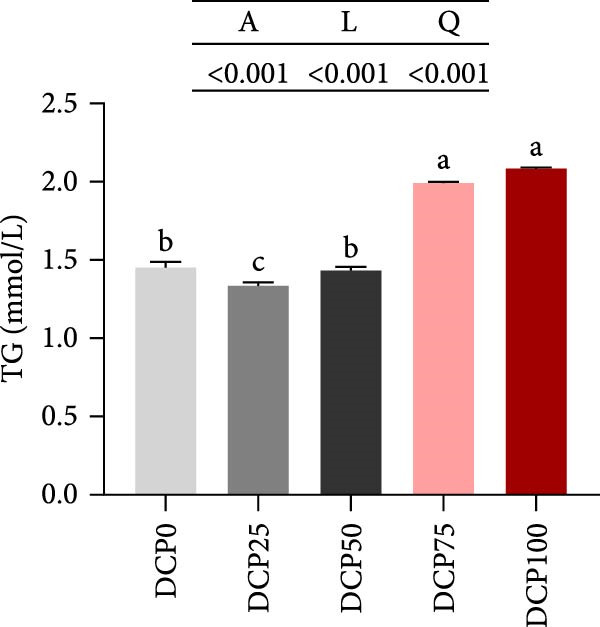
(B)

**Figure:**
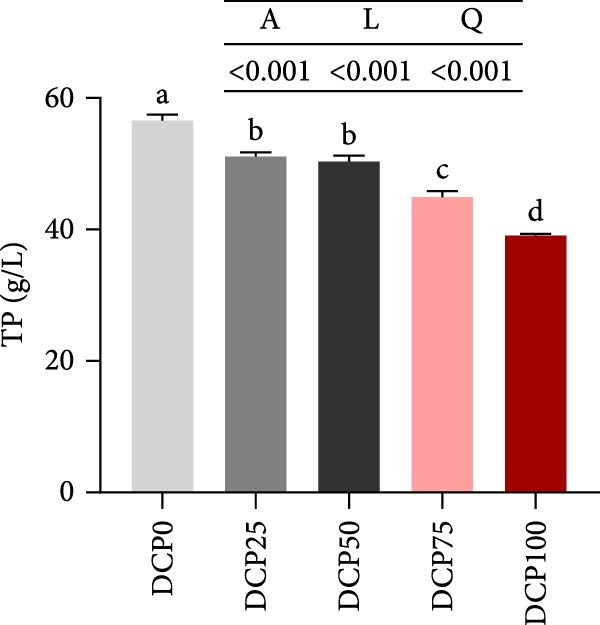
(C)

**Figure:**
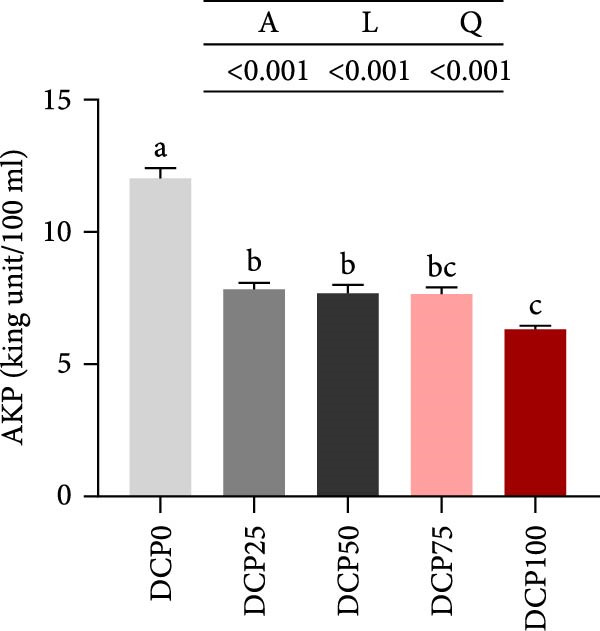
(D)

**Figure:**
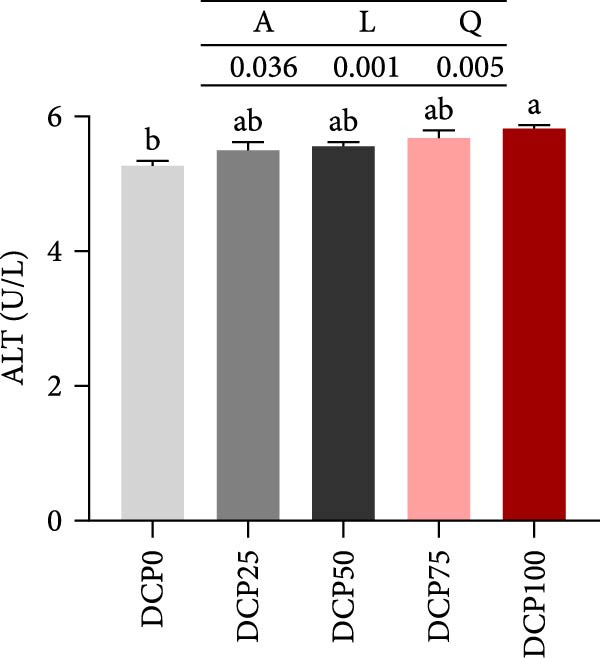
(E)

**Figure:**
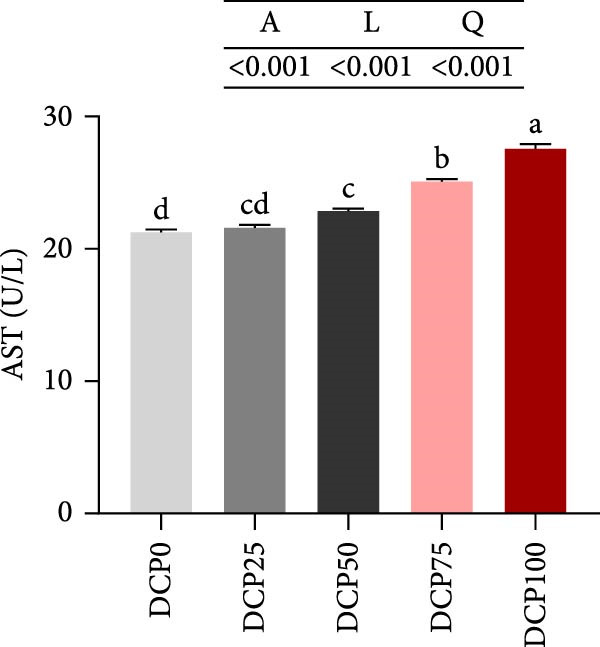
(F)

**Figure:**
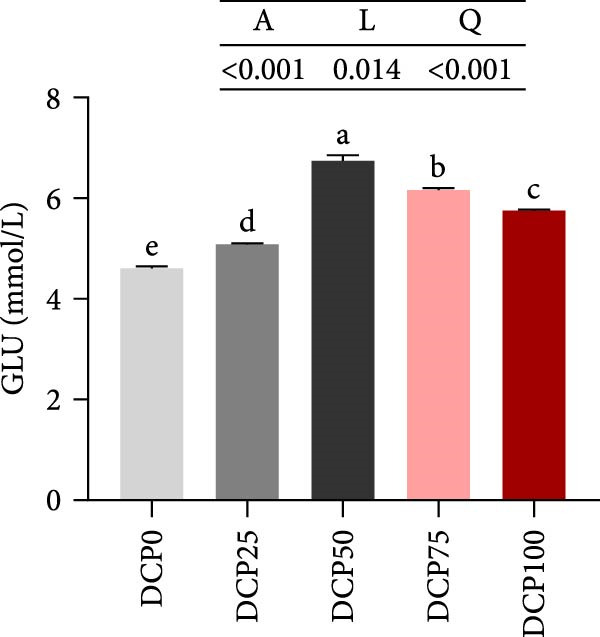
(G)

### 3.7. Expression of Genes Related to Inflammation in the Liver

With the increasing level of DCP replacement, the expression of hepatic pro‐inflammatory cytokines (*il-1β*, *il-6*, *tnf-α*, and *nlrp3*) and anti‐inflammatory cytokines (*arg1*, *tgf-β*, *il-10*, and *socs3*) shown a pattern of early decline followed by subsequent increase, corresponding to quadratic curves (Figure 7A, B). Fish fed diet with DCP replacing 100% of SBM exhibited significant upregulation of hepatic pro‐inflammatory cytokines (*il-1*β, *il-6*, and *tnf-α*) and anti‐inflammatory cytokines (*arg1* and *il-10*) compared to the control group (*p* < 0.05). In fish fed diets with DCP replacing 25%, 50%, and 75% of SBM, the pro‐inflammatory cytokine gene *nlrp3* showed significant downregulation (*p* < 0.05) (Figure 7A). Furthermore, anti‐inflammatory cytokine expression (*tgf-β* and *socs3*) exhibited significant downregulation in fish fed diets with DCP replacing 50% of SBM (*p* < 0.05) (Figure 7B).

Figure 7The expression of genes related to inflammation in liver of Hefang bream fed the experimental diets. (A) *tnf-α*, tumor necrosis factor‐α; *il-1*β, interleukin‐1β; *il-6*, interleukin‐6; *nlrp3*, *nucleotide-binding oligomerization domain-like receptor protein 3;* (B) *il-10*, interleukin‐10; *tgf-β*, transforming growth factor‐β; *arg1*, arginase1; *socs3*, suppressor of cytokine signaling 3. Values are mean ± SEM (*n* = 3). According to Tukey’s multiple comparison test, Bars with different superscript letters indicate significant differences (*p* < 0.05). Values are mean ± S.E.M. (*n* = 3). According to Tukey’s multiple comparison test, bars with same superscript letters indicate not significant differences (*p* > 0.05). A represents the *p*‐value of one‐way ANOVA; L represents the *p*‐value of linear trend; Q represents the *p*‐value of quadratic trend.

**Figure:**
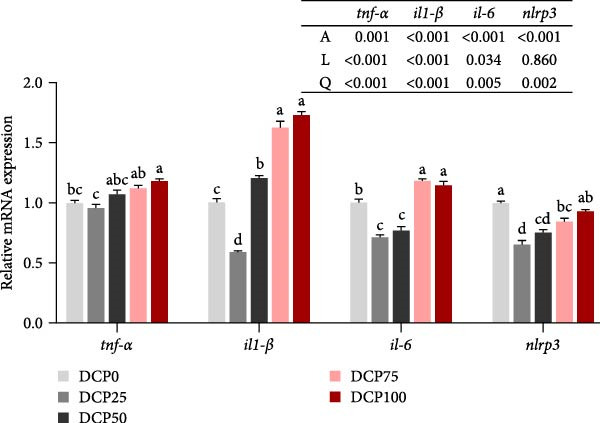
(A)

**Figure:**
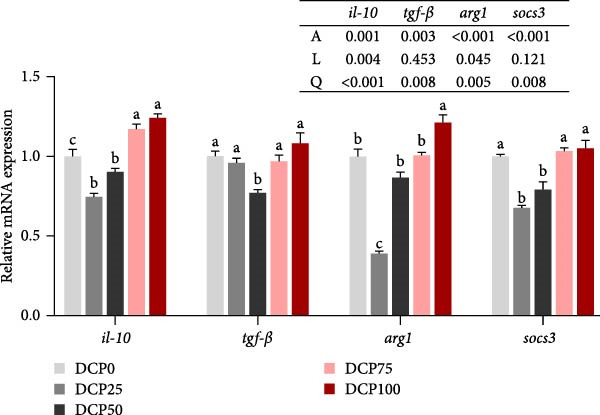
(B)

## 4. Discussion

Growth performance is a critical indicator for assessing the efficacy of protein source substitution [24–26]. In the present study, the inclusion of DCP in diets did not significantly affect the SR of Hefang bream. This indicated that DCP was safe for use in Hefang bream feed. However, the FBW and SGR of Hefang bream gradually decreased as the substitution of SBM with DCP increased, suggesting that replacing more than 50% of dietary SBM with DCP significantly impaired the growth performance of Hefang bream. This finding aligned with prior study on a large yellow croaker, in which replacing 80% of fish meal (FM) with DCP in diets induced significant reductions in SGR [11]. Meanwhile, the PER also demonstrated a declining trend. Previous studies have indicated that a reduction in PER can lead to a decrease in the SGR, ultimately resulting in diminished growth performance [27]. Likewise, high substitution levels (exceeding 60%) exerted an adverse impact on growth performance in largemouth bass (*Micropterus salmoides*) [28–30]. Research indicated that a decline in fish growth resulting from excessive DCP intake is likely due to an imbalanced amino acid profile in the diet failing to support growth requirements [31]. Moreover, previous studies demonstrate that protein substitution in aquafeeds can influence the amino‐acid profile and protein metabolism of organisms, ultimately leading to changes in growth performance [32, 33].

Previous studies have found that growth performance in fish is closely related to protein metabolic efficiency. In the present study, the whole‐fish crude protein content exhibited a significant reduction in fish fed diets with DCP replacing 75% and 100% of SBM compared to the control group. This indicated that substitution (75%–100%) could compromise protein synthesis in fish. Similarly, in a study on silver sillago (*Sillago sihama Forsskál*), the whole‐fish crude protein content showed a significant reduction when 64% of FM was substituted by DCP [34]. Also, research on sea bass (*Centropristis striata*) found that replacement of FM with low‐gossypol cottonseed meal significantly reduced fish muscle protein content when the replacement level reached 100% [35]. Meanwhile, diets deficient in essential amino acids may impair fish growth and reduce dietary protein utilization efficiency [36, 37]. Previous studies have indicated that high levels of SBM can limit fish growth performance, potentially due to insufficient lysine and methionine levels [38]. In the present study, muscle amino acid composition analysis revealed that variations in fish protein levels may be attributed to changes in muscle amino acid content. Consistent with the present study, 100% replacement of FM with CPC in diets of sturgeon (*Acipenser schrenckii*) significantly reduced whole‐body crude protein content and decreased levels of essential amino acids [39]. Tian et al. (2022) reported that complete replacement of FM with CPC in diets significantly reduced muscular threonine content of a large yellow croaker. Furthermore, changes in amino acid levels can alter the expression of genes related to protein metabolism. Studies have shown that lysine regulated the expression of *tor* and *4ebp1*, which affected protein synthesis in fish, thereby influencing protein deposition efficiency and growth performance [40]. Moreover, leucine promoted protein synthesis and deposition in juvenile white shrimp (*Litopenaeus vannamei*) by activating the *tor* signaling pathway [41]. Therefore, it is essential to analyze the alterations in genes associated with protein metabolism. In both liver and muscle, the expression of genes related to the mTOR pathway (*tor* and *s6k1*) in fish fed diets with DCP replacing 75% and 100% of SBM was significantly downregulated, while markedly upregulated the expression of *4ebp1*. These findings suggest that the observed decline in growth performance and fish body protein, resulting from high levels of SBM replacement with DCP, may be mediated through the modulation of PER and protein metabolism‐related gene expression.

Fish growth performance directly depends on their nutrient digestion and absorption capacity, which is amplified by intestinal villi elongation increasing nutrient‐intestinal contact area. Conversely, villous atrophy indicated a reduction in the absorptive capacity of intestinal nutrients [42–44]. Analysis of intestinal morphology in the present study revealed that higher substitution rates (75% and 100%) induced notable alterations in intestinal structure. With increasing dietary DCP, the lengths of intestinal villi were significantly shorter compared to the control group when substitution rates exceeded 50%, indicating that high substitution levels might adversely affect gut health. Similarly, a significant reduction in villus length was observed in pearl gentian grouper (*Epinephelus fuscoguttatus* ♀ × E. *lanceolatus* ♂) when replacing over 48% of FM with CPC [45]. Moreover, substituting more than 30% of dietary SBM protein with CPC was found to reduce both villus length and width within the intestinal tract of tilapia (*Oreochromis niloticus*) [46]. Intestinal digestive enzyme activities were next further analyzed. The reduced activity of trypsin suggested that high substitution levels compromised protein digestion and utilization in fish, potentially contributing to reduced growth performance. Similarly, a study on a large yellow croaker demonstrated that CPC replacing more than 15% of FM led to a significant reduction in trypsin activity [47]. Furthermore, a study on silver sillago showed that replacing over 32% of dietary FM with low‐gossypol cottonseed meal significantly reduced intestinal trypsin activity [34]. Overall, in the present study, high dietary DCP substitution levels led to a reduction in villus length, which may, in turn, suppress intestinal trypsin activity and ultimately impair growth performance.

The impact of high‐level replacement of SBM by DCP affected on fish not only through growth performance but also health status. Indicators such as antioxidant capacity and serum biochemical parameters serve as valuable markers for assessing the health status of fish [48–50]. Analysis of liver antioxidants, aimed at assessing potential liver injury, revealed that fish fed a diet with 100% of SBM replaced by DCP exhibited significantly reduced levels of T‐AOC, CAT activity, and GSH content compared to the control group. These findings imply that high DCP substitution level reduced the liver’s antioxidant capacity, leading to oxidative damage. Similarly, replacing over 40% of FM in diets with low‐gossypol cottonseed protein has been shown to significantly reduce intestinal SOD and CAT activities, inducing oxidative stress in hybrid grouper (*Epinephelus fuscoguttatus* ♀ *× Epinephelus lanceolatus* ♂) [51]. Further analysis of serum biochemical parameters demonstrated that ALT and AST activities increased with higher levels of DCP substitution, with both enzymes significantly elevated in fish fed the diet with DCP replacing 100% of SBM. This elevation suggested that high levels of DCP substitution induced liver damage. Supporting this finding, Zhang et al. [52] revealed that replacing more than 45% of FM with low‐gossypol cottonseed meal significantly increased serum ALT and AST activities, leading to liver injury in turbot. Besides, Chen et al. [11] demonstrated that dietary replacement of 20% FM with DCP significantly elevated serum ALT and AST activities in large yellow croaker, indicating potential hepatic impairment. Alkaline phosphatase (AKP) is classified as a lysosomal enzyme, while lysozyme serves as a crucial component of the fish’s innate immune defense. Together, AKP and lysozyme function as effective antimicrobial agents [53]. In the present study, increasing DCP substitution levels resulted in a gradual decrease in AKP activity, with fish fed diets from all substitution groups exhibiting significantly lower AKP activity. These findings hypothesized that high levels of substitution (100%) may be detrimental to fish health.

To further investigate liver injury resulting from DCP substitution, hepatic mRNA expression of inflammation‐related genes was measured. As DCP levels in the feed increased, the expression levels of hepatic pro‐inflammatory cytokines (*il-6* and *il-1*β) and anti‐inflammatory cytokines (*arg1* and *il-10*) in fish fed the diet with DCP replacing 100% of SBM were upregulated, suggesting that high substitution levels may induce inflammatory responses in the liver. Likewise, Ye et al. (2020) found that replacing more than 40% of FM in diets with CPC significantly upregulated the expression levels of *il-1*β and *il-10* in the gut of grouper, indicating induced inflammatory responses. In general, the upregulation of anti‐inflammatory cytokines prevents aberrant expression of immune responses, and the upregulation of *il-10* expression may serve as a negative feedback mechanism attempting to counteract inflammatory processes [54]. Liu et al. [55] reported that dietary replacement of 75% FM with CPC significantly suppressed hepatic expression of *tgf-β* and *il-10* in largemouth bass, suggesting a potential of hepatic inflammatory status. These findings suggested that replacing SBM with high levels of CPC appeared to suppress immune function in the gut and liver of aquatic animals.

In summary, DCP can replace 50% of SBM protein in the diet without a significant effect on the growth performance of Hefang bream. However, excessive DCP (75%–100%) in the diet significantly impaired growth performance, suppressed protein utilization efficiency, reduced digestive capacity, and induced hepatic damage in fish.

## Conflicts of Interest

The authors declare no conflicts of interest.

## Author Contributions

**Qiuxin Yan:** conceptualization, investigation, data curation, formal analysis, writing – original draft, writing – review & editing. **Xianyong Bu:** investigation, writing – review & editing. **Yongtao Liu:** investigation, validation. **Chuanwei Yao:** investigation, validation. **Zhen Wang:** methodology, formal analysis. **Manman Shi:** investigation, software. **Zhihao Zhang:** methodology, formal analysis. **Jinze Zhang:** investigation, methodology. **Junzhi Zhang:** methodology, formal analysis. **Jianlong Du:** methodology, supervision. **Yi Hu:** investigation, validation. **Yueru Li:** writing – review & editing. **Kangsen Mai:** validation, supervision. **Qinghui Ai:** writing – review & editing, funding acquisition, supervision, conceptualization.

## Funding

This investigation was supported by the National Natural Science Foundation of China (U19A2040) and the Major Science and Technology Project of Xinjiang Uygur Autonomous Region (2024A02001‐4).

## Data Availability

The data that support the findings of this study are available in the Supporting Information.

## References

[1] Gatlin D. M. , Barrows F. T. , Brown P. et al., Expanding the Utilization of Sustainable Plant Products in Aquafeeds: A Review, Aquaculture Research. (2007) 38, no. 6, 551–579, 10.1111/j.1365-2109.2007.01704.x, 2-s2.0-34248171231.

[2] Sørensen M. , Penn M. , El-Mowafi A. et al., Effect of Stachyose, Raffinose and Soya-Saponins Supplementation on Nutrient Digestibility, Digestive Enzymes, Gut Morphology and Growth Performance in Atlantic Salmon (*Salmo salar*, L), Aquaculture. (2011) 314, no. 1–4, 145–152, 10.1016/j.aquaculture.2011.02.013, 2-s2.0-79952736290.

[3] Pang A. , Xin Y. , Xie R. et al., Differential Analysis of Fish Meal Substitution With Two Soybean Meals on Juvenile Pearl Gentian Grouper, Frontiers in Marine Science. (2023) 10, 10.3389/fmars.2023.1170033, 1170033.

[4] Li H. , Hu Z. , Liu S. et al., Influence of Dietary Soybean Meal Replacement With Yellow Mealworm (*Tenebrio molitor*) on Growth Performance, Antioxidant Capacity, Skin Color, and Flesh Quality of Mirror Carp (*Cyprinus carpio* Var. Specularis), Aquaculture. (2022) 561, 10.1016/j.aquaculture.2022.738686, 738686.

[5] Ozturk E. , Darmawan A. , Ozlu S. , and Abaci S. H. , Effects of Dietary Local Hemp Seed Meal as Soybean Meal Alternative on Productive Performance, Egg Quality and Yolk Fatty Acid Composition of Laying Hens, Archives of Animal Nutrition. (2024) 78, no. 2, 178–191, 10.1080/1745039X.2024.2373485. 39047154

[6] Qu Y. , Han F. , Qiao Y. et al., Effects of Replacing Soybean Meal With Fermented Rapeseed Meal in Low-Fish-Meal Feed on the Growth, Immunity, and Gut Microbiota of Juvenile White Shrimp, *Litopenaeus vannamei* , Aquaculture. (2025) 595, 10.1016/j.aquaculture.2024.741693, 741693.

[7] Li X. , Chen S. , Sun J. et al., Partial Substitution of Soybean Meal With Faba Bean Meal in Grass Carp (*Ctenopharyngodon idella*) Diets, and the Effects on Muscle Fatty Acid Composition, Flesh Quality, and Expression of Myogenic Regulatory Factors, Journal of the World Aquaculture Society. (2020) 51, no. 5, 1145–1160, 10.1111/jwas.12671.

[8] Shi Y. , Cao X. , Zhong L. et al., Application of Sunflower Meal in Diets of on-Growing Grass Carp (*Ctenopharyngodon idellus*) and Evaluation of Enzymatic Hydrolysis, Aquaculture. (2023) 563, 10.1016/j.aquaculture.2022.738908, 738908.

[9] Wang W. , Li W. , Wu Q. et al., Isolation and Identification of a Rumen *Lactobacillus* Bacteria and Its Degradation Potential of Gossypol in Cottonseed Meal during Solid-State Fermentation, Microorganisms. (2021) 9, no. 11, 10.3390/microorganisms9112200. PMC8622920 34835326

[10] Huang X. , Hu Y. , Li Z. et al., Dephenolization Methods, Quality Characteristics, Applications, and Advancements of Dephenolized Cottonseed Protein: Review, Foods. (2025) 14, no. 4, 10.3390/foods14040628. PMC11854183 40002072

[11] Chen S. , Tang Y. , Zhang Z. et al., Replacement of Dietary Fishmeal Protein With Degossypolized Cottonseed Protein on Growth Performance, Nonspecific Immune Response, Antioxidant Capacity, and Target of Rapamycin Pathway of Juvenile Large Yellow Croaker (*Larimichthys crocea*), Aquaculture Nutrition. (2022) 2022, 10.1155/2022/8529556, 8529556. 36860446 PMC9973143

[12] Li X. , Li H. , Ji H. et al., Substitution of Soybean Meal With Cottonseed Protein Concentrate on Growth Performance, Health Status and Fish Quality of Grass Crap (*ctenopharyngodon idella*), Acta Hydrobiologica Sinica. (2023) 47, no. 2, 235–248.

[13] Wang H. , Hu X. , Zheng Y. et al., Effects of Replacing Fish Meal With Cottonseed Protein Concentrate on the Growth, Immune Responses, Digestive Ability and Intestinal Microbial Flora in *Litopenaeus vannamei* , Fish & Shellfish Immunology. (2022) 128, 91–100, 10.1016/j.fsi.2022.07.067. 35921932

[14] Gong D. , Tao M. , Wang X. et al., Studies on the Artificial Propagation of Hybrid Bream in Brackish Water, Reproduction and Breeding. (2022) 2, no. 1, 18–21, 10.1016/j.repbre.2022.02.001.

[15] Li S. , Yang X. , Fan S. et al., Comparative Analysis of Muscle Nutrient in Two Types of Hybrid Bream and Native Bream, Reproduction and Breeding. (2022) 2, no. 3, 71–77, 10.1016/j.repbre.2022.06.002.

[16] Mao Z. , Chen Y. , Cao S. et al., Effects of the Total Fish Meal Replacement by Plant Meal on Growth Performance, Nutrient Utilization and Intestinal Microbiota of Backcross F2 Derived From Blunt Snout Bream (*Megalobrama amblycephala*, ♀) × Topmouth Culter (*Culter alburnus*, ♂), Aquaculture Reports. (2024) 34, 10.1016/j.aqrep.2023.101889, 101889.

[17] Wen M. , Zhu C. , Tang Y. et al., Mechanism of Hypoxia Tolerance Improvement in Hybrid Fish Hefang Bream, Aquaculture. (2025) 599, 10.1016/j.aquaculture.2025.742199, 742199.

[18] Ren L. , Li W. , Qin Q. et al., The Subgenomes Show Asymmetric Expression of Alleles in Hybrid Lineages of *Megalobrama amblycephala* x *Culter alburnus* , Genome Research. (2019) 29, no. 11, 1805–1815, 10.1101/gr.249805.119. 31649058 PMC6836732

[19] Wu C. , Huang X. , Chen Q. et al., The Formation of a New Type of Hybrid Culter Derived from a Hybrid Lineage of *Megalobrama amblycephala* (♀) × *Culter alburnus* (♂), Aquaculture. (2020) 525, 10.1016/j.aquaculture.2020.735328, 735328.

[20] Gong D. , Xu L. , Liu Q. et al., A New Type of Hybrid Bream Derived From a Hybrid Lineage of *Megalobrama amblycephala* (♀) × *Culter alburnus* (♂), Aquaculture. (2021) 534, 10.1016/j.aquaculture.2020.736194, 736194.

[21] AOAC , Official Methods of Analysis, 1995, 16th edition., Association of Official Analytical Chemists.

[22] Folch J. , Lees M. , and Sloane Stanley G. H. , A Simple Method for the Isolation and Purification of Total Lipides From Animal Tissues, The Journal of Biological Chemistry. (1957) 226, no. 1, 497–509. 13428781

[23] Livak K. J. and Schmittgen T. D. , Analysis of Relative Gene Expression Data Using Real-Time Quantitative PCR and the 2^−ΔΔCT^ Method, Methods. (2001) 25, no. 4, 402–408. 11846609 10.1006/meth.2001.1262

[24] Zhang M. , Wang S. , Gan L. et al., Effects of Fishmeal Replacement With Eight Protein Sources on Growth Performance, Blood Biochemistry and Stress Resistance in *Opsariichthys bidens* , Aquaculture Nutrition. (2021) 27, no. 6, 2529–2540.

[25] Chen L. , Zhong J. , Shi M. et al., Effects of Replacing Fishmeal With Different Proportions of Mixed Protein Source in the Diet of Largemouth Bass (*Micropterus salmoides*), Comparative Biochemistry and Physiology D-Genomics & Proteomics. (2024) 49, 101181. 10.1016/j.cbd.2023.101181 38141372

[26] Qian Y. , Limbu S. , Qiao F. et al., Seeking the Best Alternatives: A Systematic Review and Meta-Analysis on Replacing Fishmeal With Plant Protein Sources in Carnivorous Fish Species, Reviews in Aquaculture. (2024) 16, no. 3, 1099–1126.

[27] Marchi A. , Bonaldo A. , Di Biase A. et al., Towards a Free Wild-Caught Fishmeal, Fish Oil and Soy Protein in European Sea Bass Diet Using By-Products from Fishery and Aquaculture, Aquaculture. (2023) 573, 10.1016/j.aquaculture.2023.739571, 739571.

[28] He G. , Zhang T. , Zhou X. et al., Effects of Cottonseed Protein Concentrate on Growth Performance, Hepatic Function and Intestinal Health in Juvenile Largemouth Bass, *Micropterus salmoides* , Aquaculture Reports. (2022) 23, 10.1016/j.aqrep.2022.101052, 101052.

[29] Xu G. , Wei H. , Peng D. et al., Effects of Dietary Fish Meal Replaced by Cottonseed Protein Concentrate on Growth Performance, Antioxidant Capacity, and Liver and Intestinal Health of Juvenile Hybrid Culter, Fishes. (2024) 9, no. 4, 10.3390/fishes9040127.

[30] Xu X. , Li X. , Xu Z. et al., Replacing Fishmeal With Cottonseed Protein Concentrate in Practical Diet of Largemouth Bass (*Micropterus salmoides*): Growth, Flesh Quality and Metabolomics, Aquaculture. (2024) 579, 10.1016/j.aquaculture.2023.740164, 740164.

[31] Xu Y. X. , Ding Z. L. , He X. H. , and Fei H. , Cottonseed Protein Concentrate as Fish Meal Substitution in Fish Diet: A Review, Fish Physiology and Biochemistry. (2025) 51, no. 3, 10.1007/s10695-025-01525-8. 40493267

[32] Zheng J. , Zhang W. , Dan Z. et al., Replacement of Dietary Fish Meal With *Clostridium Autoethanogenum* Meal on Growth Performance, Intestinal Amino Acids Transporters, Protein Metabolism and Hepatic Lipid Metabolism of Juvenile Turbot (*Scophthalmus maximus* L.), Frontiers in Physiology. (2022) 13, 10.3389/fphys.2022.981750, 981750. 36091361 PMC9451173

[33] Hussain S. M. , Bano A. A. , Ali S. et al., Substitution of Fishmeal: Highlights of Potential Plant Protein Sources for Aquaculture Sustainability, Heliyon. (2024) 10, no. 4, 10.1016/j.heliyon.2024.e26573. PMC10906437 38434023

[34] Liu H. , Dong X. , Tan B. et al., Effects of Fish Meal Replacement by Low-Gossypol Cottonseed Meal on Growth Performance, Digestive Enzyme Activity, Intestine Histology and Inflammatory Gene Expression of Silver Sillago (*Sillago sihama Forsskál*) 1775, Aquaculture Nutrition. (2020) 26, no. 5, 1724–1735, 10.1111/anu.13123.

[35] Anderson A. D. , Alam M. S. , Watanabe W. O. et al., Full Replacement of Menhaden Fish Meal Protein by Low-Gossypol Cottonseed Flour Protein in the Diet of Juvenile Black Sea Bass *Centropristis striata* , Aquaculture. (2016) 464, 618–628, 10.1016/j.aquaculture.2016.08.006, 2-s2.0-84982170193.

[36] Jia S. , Li X. , He W. , and Wu G. , Wu G. , Protein-Sourced Feedstuffs for Aquatic Animals in Nutrition Researchand Aquaculture, Recent Advances in Animal Nutrition and Metabolism, 2022, Springer International Publishing, 237–261. 10.1007/978-3-030-85686-1_12 34807445

[37] Molinari G. S. , Wojno M. , and Kwasek K. , Effects of Dietary Indispensable Amino Acid Deficiencies on Feed Intake in Stomachless Fish, Comparative Biochemistry and Physiology Part A: Molecular & Integrative Physiology. (2024) 298, 10.1016/j.cbpa.2024.111742, 111742. 39276852

[38] Jiang J. , Xu S. , Feng L. et al., Lysine and Methionine Supplementation Ameliorates High Inclusion of Soybean Meal Inducing Intestinal Oxidative Injury and Digestive and Antioxidant Capacity Decrease of Yellow Catfish, Fish Physiology and Biochemistry. (2018) 44, no. 1, 319–328, 10.1007/s10695-017-0437-1, 2-s2.0-85032892917. 29098470

[39] Wang C. , Zhao Z. , Lu S. et al., Physiological, Nutritional and Transcriptomic Responses of Sturgeon (*Acipenser schrenckii*) to Complete Substitution of Fishmeal With Cottonseed Protein Concentrate in Aquafeed, Biology. (2023) 12, no. 4, 10.3390/biology12040490. PMC10135981 37106691

[40] Li X. , Tang L. , Hu K. et al., Effect of Dietary Lysine on Growth, Intestinal Enzymes Activities and Antioxidant Status of Sub-Adult Grass Carp (*Ctenopharyngodon idella*), Fish Physiology and Biochemistry. (2014) 40, no. 3, 659–671, 10.1007/s10695-013-9874-7, 2-s2.0-84900316774. 24174167

[41] Wei Z. , Zhuang Y. , Liu X. et al., Leucine Promotes Protein Synthesis of Juvenile White Shrimp *Litopenaeus vannamei* Through TOR Signaling Pathway, Aquaculture. (2023) 564, 10.1016/j.aquaculture.2022.739060, 739060.

[42] Sun Z. , Tan X. , Ye H. et al., Effects of Dietary Panax Notoginseng Extract on Growth Performance, Fish Composition, Immune Responses, Intestinal Histology and Immune Related Genes Expression of Hybrid Grouper (*Epinephelus lanceolatus* ♂ × *Epinephelus fuscoguttatus* ♀) Fed High Lipid Diets, Fish & shellfish immunology. (2018) 73, 234–244, 10.1016/j.fsi.2017.11.007, 2-s2.0-85038846951. 29127028

[43] He M. , Yu Y. , Li X. et al., An Evaluation of Replacing Fish Meal With Fermented Soybean Meal in the Diets of Largemouth Bass (*Micropterus salmoides*): Growth, Nutrition Utilization and Intestinal Histology, Aquaculture Research. (2020) 51, no. 10, 4302–4314.

[44] Jiao F. , Zhang L. , Limbu S. M. et al., A Comparison of Digestive Strategies for Fishes With Different Feeding Habits: Digestive Enzyme Activities, Intestinal Morphology, and Gut Microbiota, Ecology and Evolution. (2023) 13, no. 9, 10.1002/ece3.10499. PMC10495811 37706163

[45] Chen G. , Yin B. , Liu H. et al., Effects of Fishmeal Replacement With Cottonseed Protein Concentrate on Growth, Digestive Proteinase, Intestinal Morphology and Microflora in Pearl Gentian Grouper (♀ *Epinephelus fuscoguttatus*×♂ *Epinephelus Lanceolatu*), Aquaculture Research. (2020) 51, no. 7, 2870–2884.

[46] Li W. , Wu H. , Zhang L. et al., Effects of Replacing Soybean Meal Protein With Cottonseed Protein Concentrate on the Growth Condition and Intestinal Health of Nile Tilapia (*Oreochromis niloticus*), Aquaculture Nutrition. (2021) 27, no. 6, 2436–2447.

[47] Tian S. , Wu Y. , Yuan J. et al., Replacement of Dietary Fishmeal by Cottonseed Protein Concentrate on Growth Performance, Feed Utilization and Protein Metabolism of Large Yellow Croaker *Larimichthys crocea* , Aquaculture Reports. (2022) 26, 10.1016/j.aqrep.2022.101313, 101313.

[48] Satheeshkumar P. , Ananthan G. , Kumar D. S. , and Jagadeesan L. , Haematology and Biochemical Parameters of Different Feeding Behaviour of Teleost Fishes from Vellar Estuary, India, Comparative Clinical Pathology. (2012) 21, no. 6, 1187–1191, 10.1007/s00580-011-1259-7, 2-s2.0-84870620401.

[49] Slowinska M. , Nynca J. , Cejko B. I. et al., Total Antioxidant Capacity of Fish Seminal Plasma, Aquaculture. (2013) 400, 101–104.

[50] Diler O. , Ozil O. , Bayrak H. et al., Effect of Dietary Supplementation of Sumac Fruit Powder (*Rhus coriaria* L.) on Growth Performance, Serum Biochemistry, Intestinal Morphology and Antioxidant Capacity of Rainbow Trout (*Oncorhynchus mykiss*, Walbaum), Animal Feed Science and Technology. (2021) 278, 10.1016/j.anifeedsci.2021.114993, 114993.

[51] Ye G. , Dong X. , Yang Q. et al., Low-Gossypol Cottonseed Protein Concentrate Used as a Replacement of Fish Meal for Juvenile Hybrid Grouper (*Epinephelus fuscoguttatus* ♀ × *Epinephelus Lanceolatu*s ♂): Effects on Growth Performance, Immune Responses and Intestinal Microbiota, Aquaculture. (2020) 524, 10.1016/j.aquaculture.2020.735309, 735309.

[52] Zhang X. , Zhou H. , Liu C. et al., Fishmeal Substitution With Low-Gossypol Cottonseed Meal in the Diet for Juvenile Turbot (*Scophthalmus maximus* L.): Effects on Growth, Nutrients Utilization and Haematological Responses, Aquaculture Reports. (2022) 24, 10.1016/j.aqrep.2022.101149, 101149.

[53] Gobi N. , Vaseeharan B. , Chen J.-C. et al., Dietary Supplementation of Probiotic, *Bacillus licheniformis*, Dahb1 Improves Growth Performance, Mucus and Serum Immune Parameters, Antioxidant Enzyme Activity as Well as Resistance against *Aeromonas hydrophila* in Tilapia *Oreochromis mossambicus* , Fish & Shellfish Immunology. (2018) 74, 501–508, 10.1016/j.fsi.2017.12.066, 2-s2.0-85043303529. 29305993

[54] Ye H. , Zhou Y. , Su N. et al., Effects of Replacing Fish Meal With Rendered Animal Protein Blend on Growth Performance, Hepatic Steatosis and Immune Status in Hybrid Grouper (*Epinephelus fuscoguttatus* ♀ x *Epinephelus lanceolatus* ♂), Aquaculture. (2019) 511, 10.1016/j.aquaculture.2019.734203, 2-s2.0-85067230529, 734203.

[55] Liu Y. , Lu Q. , Xi L. et al., Effects of Replacement of Dietary Fishmeal by Cottonseed Protein Concentrate on Growth Performance, Liver Health, and Intestinal Histology of Largemouth Bass (*Micropterus salmoides*), Frontiers in Physiology. (2021) 12, 10.3389/fphys.2021.764987, 764987. 34992547 PMC8724133

